# Nonlinear Hysteresis Modeling of Piezoelectric Actuators Using a Generalized Bouc–Wen Model

**DOI:** 10.3390/mi10030183

**Published:** 2019-03-12

**Authors:** Jinqiang Gan, Xianmin Zhang

**Affiliations:** 1 School of Mechanical Engineering and Electronic Information, China University of Geosciences, Wuhan 430074, China; 2 Guangdong Provincial Key Laboratory of Precision Equipment and Manufacturing Technology, School of Mechanical and Automotive Engineering, South China University of Technology, Guangzhou 510640, China; zhangxm@scut.edu.cn

**Keywords:** piezoelectric ceramics actuators, hysteresis modeling, Bouc–Wen model

## Abstract

Hysteresis behaviors exist in piezoelectric ceramics actuators (PCAs), which degrade the positioning accuracy badly. The classical Bouc–Wen (CB–W) model is mainly used for describing rate-independent hysteresis behaviors. However, it cannot characterize the rate-dependent hysteresis precisely. In this paper, a generalized Bouc–Wen (GB–W) model with relaxation functions is developed for both rate-independent and rate-dependent hysteresis behaviors of piezoelectric actuators. Meanwhile, the nonlinear least squares method through MATLAB/Simulink is adopted to identify the parameters of hysteresis models. To demonstrate the validity of the developed model, a number of experiments based on a 1-DOF compliant mechanism were conducted to characterize hysteresis behaviors. Comparisons of experiments and simulations show that the developed model can describe rate-dependent and rate-independent hysteresis more accurately than the classical Bouc–Wen model. The results demonstrate that the developed model is effective and useful.

## 1. Introduction

Piezoelectric materials are capable of undergoing reversible phase transitions as a result of voltage and pressure. Due to these special material properties, piezoelectric materials have become increasingly popular in sensors and actuators. Piezoelectric ceramics actuators (PCAs) have been employed in precision positioning systems for their large force generation, high stiffness, high resolution and fast response. However, they commonly exhibit strong hysteresis behaviors, which greatly degrade the overall positioning accuracy.

According to whether the rate of the input is considered or not, hysteresis behaviors can be divided into rate-dependent and rate-independent hysteresis behaviors. The corresponding hysteresis models can be classified into rate-dependent and rate-independent hysteresis models. Over the past few decades, great efforts have been devoted to developing hysteresis models such as the Prandtl–Ishlinskii model [1,2,3], Preisach model [4,5,6], Maxwell-Slip model [7,8], Duhem model [9,10], Polynomial-based hysteresis model [11,12], and Bouc–Wen model [13,14]. For modeling of rate-independent hysteresis, it mainly focuses on the nonlinear relationship between the amplitude of input voltage and output displacement at low input frequency or rate. However, for modeling of rate-dependent hysteresis, it needs an analysis of the nonlinear relationship between the rate of input voltage and output displacement at high input frequency or rate. Comparisons of rate-dependent and rate-independent hysteresis models reveal that the rate-independent model is just a special kind of rate-dependent model when the rate of input is low enough. Therefore, it is more difficult to develop the rate-dependent model relatively. Overall, most related literatures focused on developing rate-independent hysteresis models and few literatures paid attention to modeling of rate-dependent hysteresis.

Due to its differential equations and ability to capture an analytical form, the Bouc–Wen model has been widely applied in hysteresis modeling and compensation for piezoelectric ceramics actuators. Based on the classical Bouc–Wen (CB–W) model, Zhu and Wang [15] added a non-symmetrical formula to describe non-symmetrical hysteresis and the corresponding experiments demonstrated its validity. Fujii et al. [16] proposed an extended Bouc–Wen model by introducing a velocity sign sensitivity. To eliminate the influence of nonlinear hysteresis, Li et al. [17] presented an adaptive sliding mode control with perturbation estimation (SMCPE) based on the classical Bouc–Wen model. In addition, Liu et al. [18] proposed an adaptive neural output-feedback control based on a modified Bouc–Wen model. Lin and Yang [19] used a Bouc–Wen model to describe the hysteresis behavior and designed a hysteresis-observer based control to compensate for the piezoelectric actuator.

It should be noted that the piezoelectric actuator possesses a non-symmetrical hysteresis according to a lot of experimental research [20,21]. When the input rate is high, the non-symmetrical characteristic of the piezoelectric actuator is more serious. However, the classical Bouc–Wen model is used to describe a symmetrical hysteresis. When the input frequency or rate is low, the modeling error of the classical Bouc–Wen model is not large. However, when the input frequency or rate is high, its modeling error is large. Therefore, it can be found that the classical Bouc–Wen model is mainly used to characterize the rate-independent hysteresis behavior and modeling, but cannot characterize the rate-dependent hysteresis behavior precisely though it is a rate-dependent hysteresis model according to traditional classifications. The modeling accuracies of published hysteresis models are not high enough. Furthermore, due to the existence of many parameters and differential equations, it is a hard task to identify the parameters of hysteresis models.

In our previous work [22], we have developed an enhanced Bouc–Wen model by introducing input frequency. But there is a limitation that the developed model cannot be applied when the input frequency is unknown. To solve the problems above, this paper proposed a generalized Bouc–Wen (GB–W) model by introducing relaxation functions in the classical Bouc–Wen model, which can characterize both rate-independent and rate-dependent hysteresis behavior for piezoelectric ceramics actuators precisely. A lot of experiments are conducted in advance to characterize hysteresis behaviors and subsequently the relaxation functions are determined cautiously based on these experimental characteristics. The generalized Bouc–Wen (GB–W) model doesn’t have the aforementioned limitation and can be widely applied. In addition, the nonlinear least squares method through MATLAB/Simulink is used to identify the corresponding parameters of hysteresis models. Both simulations and experiments finally demonstrate the validity of the developed model. Therein, the classical Bouc–Wen model is set as a comparison. The rest of this paper is arranged as follows: Section 2 gives the descriptions of the classical Bouc–Wen model. In Section 3, the generalized Bouc–Wen model is presented. Section 4 gives the experimental validation of results and discussion. Finally, conclusions are drawn in Section 5.

## 2. Classical Bouc–Wen Model

The Bouc–Wen model was initially applied for nonlinear vibrational mechanics. With the rapid development of smart actuators, it was gradually used to describe nonlinear hysteresis for piezoelectric actuators. The hysteresis curve can be considered as the superposition of a linear component X(t) and a hysteretic component h(t). The classical hysteretic Bouc–Wen model as a nonlinear system is described as follows:

(1) y(t)=X(t)+h(t)=k·u(t)+h(t)

(2) $$\dot{h}(t)=\alpha \dot{u}(t)-\beta \dot{u}(t){\left|h(t)\right|}^{n}-\gamma \left|\dot{u}(t)\right|{\left|h(t)\right|}^{n-1}h(t)$$

where u(t) is the input voltage and y(t) is the output displacement. k, α, β, γ and *n* are the model parameters, which decide the shape of hysteresis curves. In order to simplify the model, *n* is usually set as 1 and the hysteretic components is expressed by

(3) $$\dot{h}(t)=\alpha \dot{u}(t)-\beta \dot{u}(t)\left|h(t)\right|-\gamma \left|\dot{u}(t)\right|h(t)$$

## 3. Generalized Bouc–Wen Model

**Figure 1:**
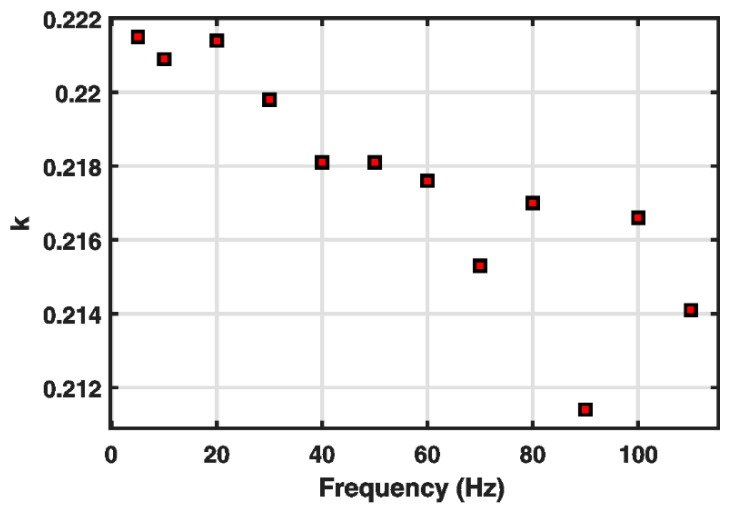
Variations of k under u(t)=5sin(2πft)+5 at different frequencies.

**Figure 2:**
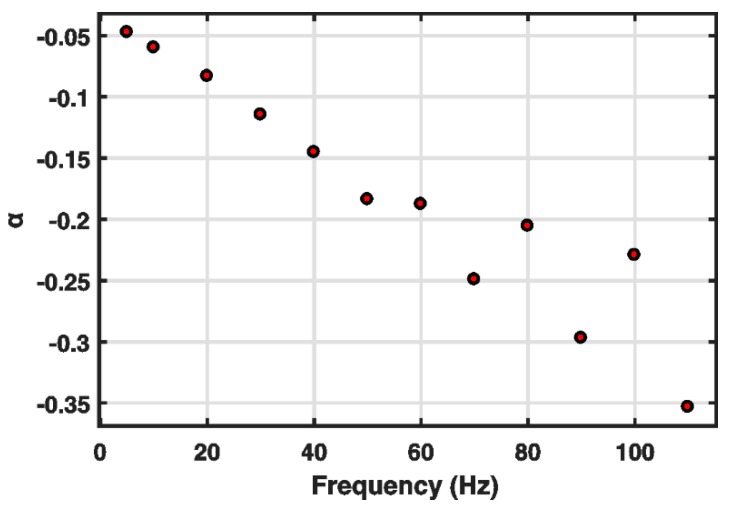
Variations of α under u(t)=5sin(2πft)+5 at different frequencies.

To analyze the performance of the classical Bouc–Wen model in detail, some efforts were devoted to research on the characteristics of its parameters. The variations of its parameters k and α at different frequencies of the input are shown in Figure 1 and Figure 2, respectively. Table 1 gives the detailed values of parameters of the classical Bouc–Wen model at different frequencies. The parameters were identified by the nonlinear least squares method through MATLAB/Simulink, which will be introduced in detail in the next part. The identified results based on experimental data clearly reveal that the parameters k and α both decrease with the increase in frequency. Such frequency dependence of the parameters k and α cannot be described by the classical Bouc–Wen model. The parameters of the classical Bouc–Wen model are fixed constants, which cannot characterize their change trend with the increase in frequency. To some degree, thus, it can be concluded that the classical Bouc–Wen model cannot describe rate-dependent hysteresis behaviors.

### 3.1. Formulation of the Generalized Bouc–Wen Model

The results above show that the classical Bouc–Wen model cannot describe rate-dependent hysteresis behaviors precisely. It is resulted by a non-symmetrical hysteresis of piezoelectric actuators while the classical Bouc–Wen model is a symmetrical model. Therefore, the classical Bouc–Wen model should be redefined to solve this problem. However, it should be noted that the redefined model should possess the capacity to describe both rate-independent and rate-dependent hysteresis, which is the purpose of this work.

How to formulate a generalized hysteresis model of piezoelectric actuators is still a hard task and some researchers have made some contributions, such as, Al Janaideh et al. [23] presented a generalized P-I model with relaxation functions to characterize rate-dependent hysteresis behaviors. Mayergoyz [24] proposed a generalized Preisach model of hysteresis by introducing a generalized density function. Based on the experimental dynamic characteristics and researchers’ experiences above, the generalized Bouc–Wen model is thus formulated upon integrating relaxation functions $k(v(t),\dot{v}(t))$ and $\alpha (v(t),\dot{v}(t))$, such as

(4) $$y(t)=X(t)+h(t)=k(u(t),\dot{u}(t))\cdot \dot{u}(t)+h(t)$$

(5) $$\dot{h}(t)=\alpha (u(t),\dot{u}(t))\cdot \dot{u}(t)-\beta \dot{u}(t)\left|h(t)\right|-\gamma \left|\dot{u}(t)\right|h(t)$$

where $k(u(t),\dot{u}(t))$ and $\alpha (u(t),\dot{u}(t))$ are relaxation functions of current input u(t) and its rate of $\dot{u}(t)$ and $k(u(t),\dot{u}(t))$ is a positive function. It should be noted that the generalized Bouc–Wen model could describe rate-independent hysteresis behaviors at low input frequency. Therefore, $k(u(t),\dot{u}(t))$ and $\alpha (u(t),\dot{u}(t))$ should converge to fixed constants when the input frequency is low. Based on the characteristics above, the relaxation functions can be defined by inducing the exponential function, such as

(6) $$k(u(t),\dot{u}(t))=pe^{-q\dot{u}(t)}$$

(7) $$\alpha (u(t),\dot{u}(t))=\varepsilon e^{\delta \left|\dot{u}(t)\right|}$$

where p≥0, q≥0, ε, δ, β and γ are constants. From the expressions of the relaxation functions, it can be found that when the input frequency is very low, such that $\dot{u}(t)≅0$, the relaxation functions converge to fixed constants, such that $k(u(t),\dot{u}(t))≅p$ and $\alpha (u(t),\dot{u}(t))≅\varepsilon$. Thus, it can describe the rate-independent hysteresis model the same as the classical Bouc–Wen model.

Compared with the classical Bouc–Wen model, the parameters $k(u(t),\dot{u}(t))$ and $\alpha (u(t),\dot{u}(t))$ of the proposed model values vary with the rate of input, which are not fixed constants any more. Furthermore, the parameters $k(u(t),\dot{u}(t))$ in the rising hysteresis curves at the same input are different from that in the decreasing hysteresis curves. The characteristics above form the non-symmetrical hysteresis of the proposed model.

### 3.2. Properties of the Generalized Bouc–Wen Model

**Figure 3:**
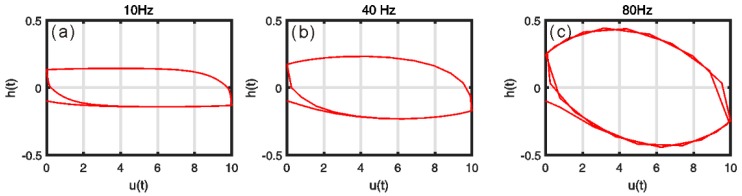
Variations of the component h(t) under u(t)=5sin(2πft)+5: (**a**) f = 10 Hz; (**b**) f = 40 Hz; (**c**) f = 80 Hz.

This section will focus on analyses of the properties of the generalized Bouc–Wen model. First, the characteristics of the hysteretic component h(t) should be analyzed based on simulations. Figure 3 shows the relationship between h(t) and input frequency f under input voltage u(t)=5sin(2πft)+5. The corresponding parameters of the generalized Bouc–Wen model are set as p=0.2107, $q=1.189\times 10^{-5}$, ε=−0.1331, $\delta =5.2622\times 10^{-4}$, β=5.3743 and γ=6.4698. The results clearly reveal that the width of the hysteretic component h(t) increases monotonically with the increase in the rate of input.

**Figure 4:**
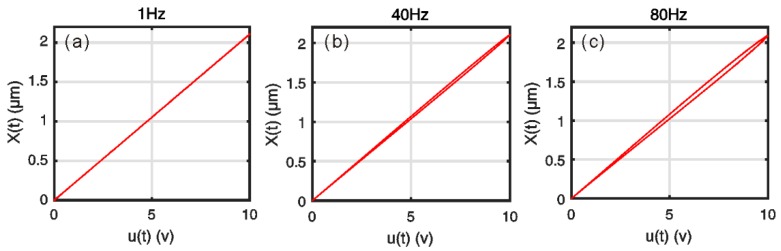
Variations of the component X(t) under u(t)=5sin(2πft)+5: (**a**) f = 10 Hz; (**b**) f = 40 Hz; (**c**) f = 80 Hz.

Figure 4 shows the relationship between the component X(t) and input frequency f. The results reveal that the component X(t) is still nearly linear with input voltages at low frequencies. But the curves of the component X(t) have a hysteresis loop with the increase in frequencies, which shows non-symmetrical characteristics.

### 3.3. Parameters Identification

Due to the existence of derivation and more parameters, it is not easy to identify the Bouc–Wen model for most researchers. In this study, the objective function F is expressed by

(8) $$F=Min\sum_{i=1}^{n}f^{2}(u)$$

with

(9) $$f(u)=y_{i}-y_{i}{}^{HM}$$

(10) $$y_{i}{}^{HM}=X(iT)+h(iT)=pe^{-q\dot{u}\left(iT\right)}\dot{u}(iT)+h(iT)$$

(11) $$\dot{h}(iT)=\varepsilon e^{\delta \left|\dot{u}(iT)\right|}\cdot \dot{u}(iT)-\beta \dot{u}(iT)\left|h(iT)\right|-\gamma \left|\dot{u}(iT)\right|h(iT)$$

where *n* is the total number of samples, *T* is the sampling period, i=1,2,3⋯n is the ith sampling period, $y_{i}{}^{HM}$ and $y_{i}$ are the predicted output by hysteresis model and experimental displacements of piezoelectric actuators, respectively. According to these equations above, it can be concluded that f(u) is a nonlinear function of the parameters p, q, ε,δ,β and γ. It is a nonlinear least squares problem. It is better to choose the nonlinear least squares method instead of the least squares method to identify parameters of the proposed model in our study.

**Figure 5:**
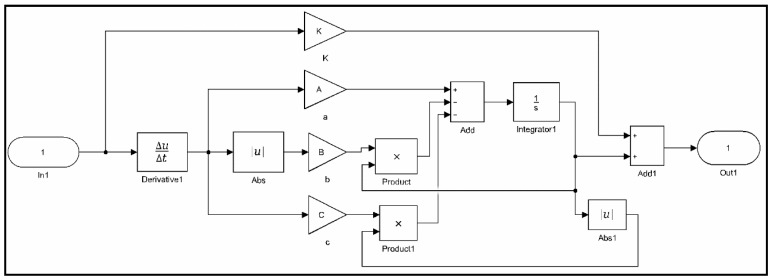
Classical Bouc–Wen (CB–W) model implemented with Matlab/Simulink.

**Figure 6:**
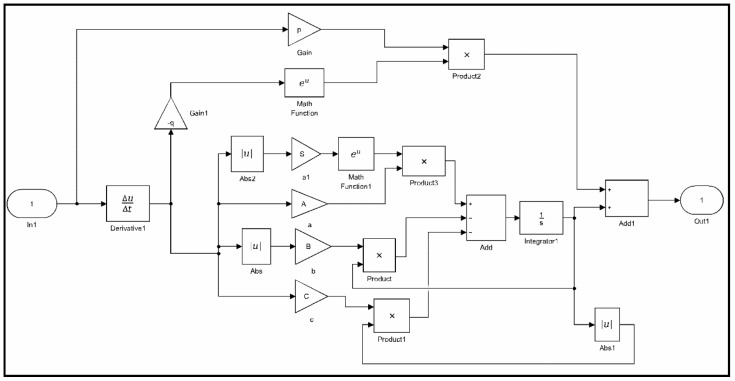
Generalized Bouc–Wen (GB–W) model implemented with MATLAB/Simulink.

In this paper, the nonlinear least squares method using the Trust-Region-Reflective algorithm is presented to identify the parameters of classical and generalized Bouc–Wen models. This method uses the nonlinear least squares function for optimization through the MATLAB/Simulink Optimization Toolbox. The corresponding identification steps of the nonlinear least squares method is carried out offline as follows:

(1) Data collection: Experimental data including output displacements and input voltages for piezoelectric actuators are obtained and recorded.
(2) Model implementation: Classical and generalized Bouc–Wen models are implemented using the MATLAB/Simulink block as shown in Figure 5 and Figure 6, respectively.
(3) Parameter estimation: The Trust-Region-Reflective algorithm is used to identify the parameters of hysteresis models based on experimental data.
(4) Validation: Comparison of the measured and simulation results predicted by hysteresis models are shown, and the corresponding modeling errors are plotted.

In traditional identification methods, the identified procedure is usually a long and complex work with many equations and steps. For example, in reference [15], Zhu and Wang added a non-symmetrical formula based on the classical Bouc–Wen model to describe non-symmetrical hysteresis. The parameters for the linear and hysteresis components are separately identified by utilizing the final value theorem of the Laplace transform and the least squares method, respectively. However, the nonlinear least squares method using the Trust-Region-Reflective algorithm in this study is presented to identify all parameters for the linear and hysteresis components at the same time, which can simplify the identification procedure. In addition, all operations are conducted by using the MATLAB/Simlink optimization tools and there are no complex algorithms needed to write, which can simplify the identification procedure further. Furthermore, there are just four steps above and the whole computing time is controlled in several minutes. Therefore, the nonlinear least squares method can quickly and simply identify the parameters of hysteresis models. Last but not least, this method can be expanded to apply to other complex model identifications, which is useful and meaningful undoubtedly.

## 4. Experimental Validation Results and Discussion

### 4.1. Experimental Setup

**Figure 7:**
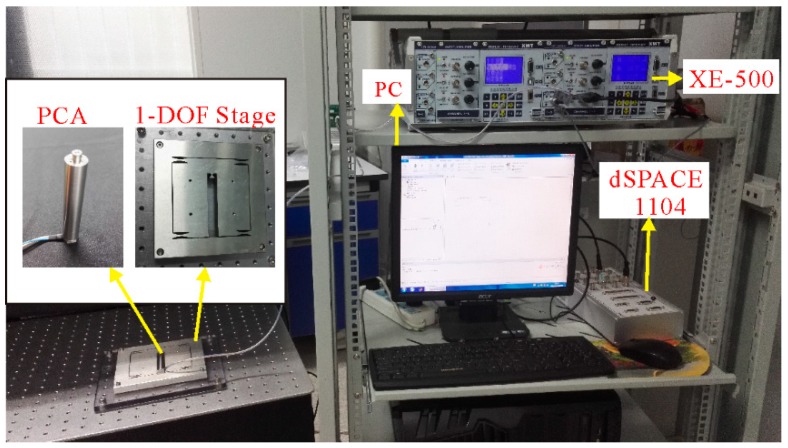
Experimental setup.

Experiments were conducted to establish the magnitude of the nonlinearity in the output of the piezoelectric actuator. The experimental setup, as shown in Figure 7, employs a 1-DOF compliant mechanism stage to produce linear motion. The stage is actuated by a stack piezoelectric ceramic actuator (Coremorrow PST 150/7/60VS12, Harbin, China), whose nominal displacement was 60 μm for the maximum input voltage of 150 V. The actuator is a preloaded piezoelectric translator manufactured by Piezomechanik in Germany and made of PZT (Pb-based Lanthanum-doped Zirconate Titanates), whose detailed information is shown in Table 2. This actuator is the superposition of the multilayer piezoelectric ceramic sheets together. The stage was set up on an optical table for vibration isolation and experiments were carried out in a clean room to minimize external noise sources.

The position of the moving stage was simultaneously measured by the strain gauge position sensor (SGS), which is included in the piezoelectric ceramic actuator. To achieve the real-time control, the dSPACE-DS1104 rapid prototyping controller board equipped with a 16-bit analogue-to-digital converter (ADC) and 16-bit digital-to-analogue converter (DAC) was used as the real-time control system. The output voltage signals were amplified by an amplifier included in XE-500 controller, which provided excitation voltage for the piezoelectric actuator from −20 V to 150 V. The measured displacement signals were obtained by a signal conditioner included in the XE-500 controller. Both the input voltage and output displacement signals were stored by a computer. The control interface is performed by the Control Desk 5.0-dSPACE and MATLAB/Simulink.

### 4.2. Results

To demonstrate the performance of the generalized Bouc–Wen (GB–W) model for the piezoelectric actuator, hysteresis behaviors in the piezoelectric actuator were measured and the classical Bouc–Wen (CB–W) model was constructed for comparison. In this case, we conducted two groups of experiments to demonstrate the effectiveness of characterizing rate-dependent and rate-independent hysteresis, respectively.

In the first group of experiments, we measured the outputs of the piezoelectric actuators under excitation voltage signals u(t)=5sin(2πft)+5(f=5,10,20,40,60,80,90,100,110) to demonstrate the effectiveness of the GB–W model to characterize rate-dependent hysteresis behaviors. The measured data of excitation signal at 110 Hz is initially adopted to identify the parameters of both generalized and classical Bouc–Wen models. The identified parameters of the generalized Bouc–Wen model are p=0.2107, $q=1.189e\times 10^{-5}$, ε=−0.1331, $\delta =5.2622\times 10^{-4}$, β=5.3743 and γ=6.4698 Meanwhile, the corresponding parameters of the classical Bouc–Wen model k=0.2141, α=−0.3534, β=3.1034 and γ=3.7229. It must be noted that the input frequency should be controlled under 150 Hz to avoid a high dynamic force of encapsulated stack piezoelectric ceramics for security protection. Therefore, the nine groups of experiments need to follow this rule.

**Figure 8:**
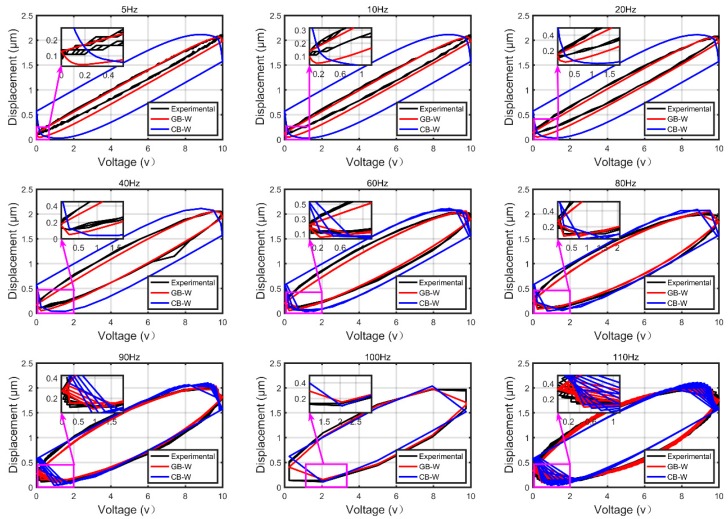
Comparisons of the measured and simulation results predicted by the GB–W model and classical CB–W model.

**Figure 9:**
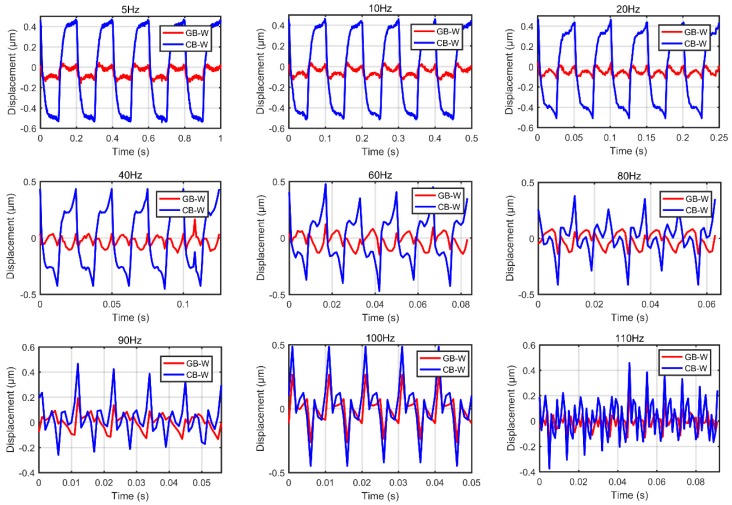
Rate-dependent modeling errors of the GB–W model and classical CB–W model.

Figure 8 shows comparisons of the measured and simulation results predicted by the generalized and classical Bouc–Wen model. The black, green and red lines represent the experimental data, and the generalized Bouc–Wen model, respectively. It can be found that the simulation results predicted by the generalized Bouc–Wen model agree better with the experimental data than that of the classical Bouc–Wen model. Figure 9 shows the corresponding modeling errors of these models. It is clearly shown that the modeling errors of the generalized Bouc–Wen model is much smaller than that of the classical Bouc–Wen model.

**Figure 10:**
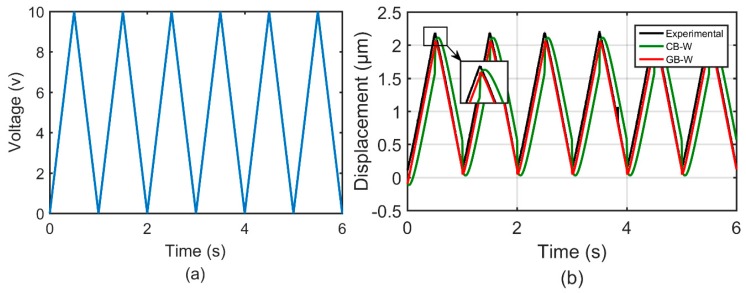
Time histories of: (**a**) A waveform of input excitation signal with the amplitude of 10 and (**b**) the measured and simulation results predicted by the GB–W model and CB–W model.

**Figure 11:**
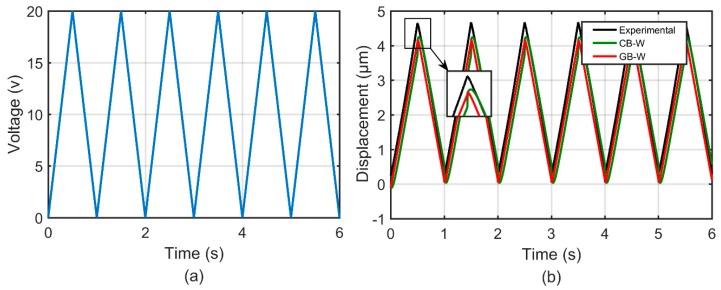
Time histories of: (**a**) A waveform of input excitation signal with the amplitude of 20 and (**b**) the measured and simulation results predicted by the GB–W model and CB–W model.

In the second group of experiments, two different waveforms of input excitation signals with the amplitude of 10 and 20, respectively, were adopted to actuate the 1-DOF compliant mechanism. These experiments are used to demonstrate the effectiveness of the GB–W model to characterize rate-independent hysteresis behaviors. The parameters of the GB–W model and CB–W model remained the same with the first group of experiments. Figure 10 and Figure 11 show comparisons of the measured and simulation results predicted by the GB–W model and CB–W model. It can be found that the predicted results by the GB–W model agree better with the measured than that by the CB–W model.

To qualify modeling errors reasonably, the root-mean-square error $e_{rms}$ (RMSE) and relative root-mean-square error δ (RRMSE) are introduced in this paper as follows:

(12) $$e_{rms}=\sqrt{\frac{1}{T}\int_{0}^{T}{\left|y(t)-y_{d}(t)\right|}^{2}dt}$$

(13) $$\delta =\frac{e_{rms}}{\max(y_{d}(t))}\times 100\%$$

where y(t) and $y_{d}(t)$ are the simulated and measured displacements, respectively, T is the total time.

The detailed modeling errors are shown in Table 3 and Table 4. The max displacement is 2.105 μm in the first group of experiments. According to it, in the first group of experiments, RMSE and RRMSE of the GB–W model under excitation signal at 5 Hz are 0.0742 μm and 3.52%, which are reduced by 81.5% compared with that of the CB–W model. When the frequency increases to 100 Hz and 110 Hz, the modeling errors (RMSE and RRMSE) of the GB–W model are reduced by nearly 42.1% and 56.47%, respectively. The results clearly reveal that the generalized Bouc–Wen model can predict the output of rate-dependent hysteresis curves of piezoelectric actuators more precisely. In the second group of experiments, the max measured displacements under two waveforms of input excitation signal are 2.215 μm and 4.676 μm, respectively. RMSE and RRMSE of the GB–W model under a waveform of input excitation signal with the amplitude of 10 are 0.1315 μm and 5.94%, respectively, which are reduced by 70.8% compared with that of the CB–W model. In the other experiment with the amplitude of 20, the rate-independent modeling errors of the GB–W model are still smaller and reduced by 28.9% compared with that of the CB–W model. The results above clearly reveal that the GB–W model can also predict the output of rate-independent hysteresis curves of piezoelectric actuators more precisely. According to the above analyses, it is reasonable to believe that the GB–W model is effective and can characterize both rate-dependent and rate-independent hysteresis behaviors more precisely than the CB–W model.

### 4.3. Discussion

The classical Bouc–Wen model is mainly used to characterize the rate-independent hysteresis, but cannot characterize the rate-dependent hysteresis precisely. Compared with the classical Bouc–Wen model, the generalized Bouc–Wen model integrates relaxation functions. $k(u(t),\dot{u}(t))$ and $\alpha (u(t),\dot{u}(t))$ based on the traditional fixed model parameters k and α. The relaxation functions are determined based on experimental characteristics instead of random imaginations and make model parameters closely related to the rate of input $\dot{u}(t)$. Therefore, the proposed model can theoretically describe the rate-dependent hysteresis behaviors more precisely and the experimental and simulation results demonstrate its effectiveness. In addition, the experimental and simulation results also show that the generalized Bouc–Wen model can describe the rate-independent hysteresis behaviors more precisely than the classical Bouc–Wen model. So it can be concluded that the generalized Bouc–Wen model can characterize both rate-dependent and rate-independent hysteresis behaviors.

The enhanced Bouc–Wen model in our previous work [22] is closely related to the input frequency f and cannot be used to describe the rate-dependent hysteresis behaviors when the frequency is unknown. However, the generalized Bouc–Wen model is closely related to the rate of input $\dot{u}(t)$ and can be widely used to describe the rate-dependent hysteresis behaviors without limitations. This is the main advantage of the developed model.

Compared with other existing models such as the Prandtl–Ishlinskii model and Preisach model, which are rate-independent models, the proposed model is a rate-dependent model. In addition, the proposed model has differential equations and the ability to capture an analytical form, which can provide more convenience for hysteresis compensation control. So the developed model has a broader application prospect in hysteresis modeling and compensation controls.

## 5. Conclusions

In this paper, a generalized Bouc–Wen model is established to characterize both rate-independent and rate-dependent hysteresis behaviors by introducing relaxation functions in the classical Bouc–Wen model. The corresponding parameter was identified by the nonlinear least squares method through Matlab/Simulink. The validity of the developed model is demonstrated by a number of experiments. Comparing the predicted data of the generalized Bouc–Wen model with the experimental data revealed reasonably good agreements. The results showed that the developed model can describe rate-dependent and rate-independent hysteresis behaviors more precisely than the classical Bouc–Wen model. The modeling errors of the generalized Bouc–Wen model can be reduced greatly.

## Figures and Tables

**Table 1:** Identified parameters of the classical Bouc–Wen (CB–W) model at different frequencies.

Frequency (Hz)	k	α	β	γ
5	0.2215	−0.0473	0.0847	0.4477
10	0.2209	−0.0598	0.2629	0.6506
20	0.2214	−0.0832	0.7183	1.1444
30	0.2198	−0.1146	1.5761	2.0662
40	0.2181	−0.1453	2.8847	3.4124
50	0.2181	−0.1838	2.0272	2.6029
60	0.2176	−0.1877	2.4755	3.0932
70	0.2153	−0.2492	4.0013	4.6221
80	0.2170	−0.2055	2.9687	3.5753
90	0.2114	−0.2970	5.9981	6.6169
100	0.2166	−0.2293	3.6008	4.2298
110	0.2141	−0.3534	3.1034	3.7229

**Table 2:** Information about the piezoelectric ceramics actuator (PCA).

Type	PST 150/7/60VS12
Material	PZT
Length [mm] ±0.3	64
Nominal Thrust/tension [N]	1800/300
Electrical capacitance [μF] ±20%	5.4
Resonant frequency [kHz]	15
Stiffness [N/μm] ±20%	15
Nominal Stroke [μm] ±15%	60

**Table 3:** Rate-dependent modeling errors of the GB–W model and CB–W model.

Frequency (Hz)	GB–W Model		CB–W Model	
	RMSE (μm)	RRMSE (%)	RMSE (μm)	RRMSE (%)
5	0.0742	3.52	0.4015	19.07
10	0.0650	3.08	0.3959	18.81
20	0.0700	3.33	0.3420	16.25
40	0.0316	1.50	0.2595	12.32
60	0.0697	3.31	0.2110	10.02
80	0.0718	3.41	0.1608	7.64
90	0.0679	3.23	0.1495	7.10
100	0.1264	6.00	0.2183	10.37
110	0.0673	3.20	0.1546	7.34

**Table 4:** Rate-independent modeling errors of the GB–W model and CB–W model.

Amplitude	GB–W Model		CB–W Model	
	RMSE (μm)	RRMSE (%)	RMSE (μm)	RRMSE (%)
10	0.1315	5.94	0.4510	20.36
20	0.3869	8.27	0.5441	11.63
